# Supplementary material for: Impacts of Climate Change Interacting Abiotic Factors on Growth, aflD and aflR Gene Expression and Aflatoxin B1 Production by Aspergillus flavus Strains In Vitro and on Pistachio Nuts
Source: Toxins (Basel). 2021 May 28;13(6):385. doi: 10.3390/toxins13060385 (PMC8228473; doi:10.3390/toxins13060385)
Supplement: Supplementary file 1 [file toxins-13-00385-s001.zip › toxins-1178230-supplementary checked.pdf]

Supplementary Materials: Impacts of Climate Change Interacting Abiotic Factors on Growth, *aflD* and *aflR* Gene Expression and Aflatoxin B_1 Production by *Aspergillus flavus* Strains *in vitro* and on Pistachio Nuts

Table S1. Sequencing results of isolated strains and type strain using ITS1 & 2 and ITS3 & 4 primer pairs for molecular identification of strains AB3 and AB10. * Type strain from the Agricultural Research Services Laboratories of the US Department of Agriculture USDA, New Orleans).

Strain ID	GenBank ID		Genus	Species	Similarity (%)
	ITS1 & 2	ITS 3 & 4			
NRRL 3357 *	M1204.653	BP4	*Aspergillu*	*flavus*	100/99
AB3	A4S3_13	SCAU-F-142	*Aspergillu*	*flavus*	100/98
AB10	M1204.653	LPSC 1183	*Aspergillu*	*flavus*	100/99
